# From Geo. Watt, Xenia

**Published:** 1882-10

**Authors:** Geo. Watt

**Affiliations:** Xenia, Ohio


### CORRESPONDENCE.

XENIA, OHIO, September 11, 1882.

*Editor* of DENTAL REGISTER: Did you ever stop?—yes; stop is the word—for we all know your mind is ever on the wing, and you can't take in a stray idea, or a wandering thought, unless you *stop* your present mental effort. To start again; did you ever stop to think that the locomotion of the frog is typical or emblematical of all progress, in life, in science, or in civilization? You well know that "the frog's the scientifickest of nature's handiwork; he neither walks, nor runs nor creeps, but goes it with a jerk. Common meter, sing. We are born on the principle of pressure and rest. Were natal physiology different, continued force without the rest, all babes would be dead born, and they would be deceased orphans as well, for there would be a dead mother beside each one. But it is as it is, and nobody wants it more so—would prefer it less so, not so often, and a little easier, perhaps. Will it not be a blessed time if the human mother shall become so healthy and vigorous that her now great trial, called *labor*, may be properly called *play*.

But everywhere the frog's motion is manifest. We work and then we sleep, or is the phrase improved by reversal in accordance with the thought—

"The rest is mother of the toil."

History teaches that the progress of civilization is in full accord with the principle now set forth. But see how the progress of our profession vindicates the same idea. Subjects were discussed when you and I were young in dentistry, and though the discussion was by no means exhaustive, they were laid aside for a time. They received their pressure, and are taking the corresponding rest. As an illustration, the grafting of crowns on the natural roots received earnest attention long ago. Some of the best modes ever suggested were the offspring of your own brain, born too, in accordance with frog locomotion—experiments made in time snatched from busy days, they being made possible because storms, or something else, kept patients from engagements. If the old Xenia laboratory could talk, and were inclined to gossip, what tales it could tell of two boys, much more nearly equal in age than in size—two boys so intent on discovery that for hours they would be so absorbed in thought as to be as speechless as the lamps that lit up the scenes of their midnight researches. But we all tire of monotony, and these subjects were laid aside, only partially solved. Again and again has pressure been applied to some of them, and, now, a new generation has sprung up and are pressing forward their investigations, some of them quite unconscious that they are not genuine pioneers in their present pathways. Only a few weeks ago one of our younger professional brethren, whom we both highly esteem, and who is worthy of our highest estimation, wrote me that he had just learned, for the first, that I had written on the subject of amalgam fillings. As he had been trying some experiments similar to those we tried more than a quarter of a century ago, and had found similar results, he was all the more gratified with our agreement. This subject of amalgam is one that got a long rest, but has come to the front again in some localities. And if we were together in some place as retired as was our good old laboratory, I would ask you if you candidly believe that the present discussions go much deeper into the subject than did the investigations of those good old days. But I'll not ask you through the REGISTER. That wouldn't be fair.

Reflex influences from the teeth are pressed again into prominent consideration, after at least a partial rest; and it will take hard pressure to exhaust matters here. Any ambitious young dentist, who feels like tackling the subject; has our full consent that he shall do so, and an assurance that he will not, in one lifetime, find out too much about it. The fear of overdoing is sometimes oppressive, but he can afford to rest easy between efforts here.

The allusion to our old Xenia laboratory calls up a long, serious train of thoughts. Our laboratory was emphatically a part of our office; and did you ever think that probably no other dental office in the world has done so many things for the profession? It may be, however, that many, or even most, of these things have not been well done. But as they are, they are, and for a long time have been before the profession. At least four college professors have emanated from that old office, two of them having been repeatedly elected. Two journalistic editors have followed suit; and nearly half a score of prominent writers have received, more or less of their professional training from it. That old laboratory gave the profession two safety alcohol lamps for soldering purposes; in it was made the first warm air syringe, for drying cavities; it gave, through Dr. S. S. White, the first artificial teeth with the first bicuspid beveled so as to avoid the abrupt shoulder; it gave the cup-shaped corundum wheel; it introduced and made the first banded work in mechanical dentistry; it made the separable extracting screw, superseding the screw-forceps, (both useless now); and we believe it furnished the first really crystalized gold for filling, but will not insist on that. But we can not recall half the good deeds of the good old laboratory. To us it it was a sacred place.

But, I intended to give you but a page; and just look! Well, you can leave out some, and better, all of it. And why didn't I write it for the *Journal?* My dear sir, the *Journal* is full; and besides, it is not sufficiently dignified for the *Journal.*

Truly yours, GEO. WATT.

